# Association of Time-Based Billing With Evaluation and Management Revenue for Outpatient Visits

**DOI:** 10.1001/jamanetworkopen.2022.29504

**Published:** 2022-08-31

**Authors:** Tyler J. Miksanek, Samuel T. Edwards, George Weyer, Neda Laiteerapong

**Affiliations:** 1 Biological Sciences Division, Pritzker School of Medicine, University of Chicago, Chicago, Illinois; 2 Division of General Internal Medicine and Geriatrics, Oregon Health & Science University, Portland; 3 Section of General Internal Medicine, Veterans Affairs Portland Health Care System, Portland, Oregon; 4 Biological Sciences Division, Department of Medicine, University of Chicago, Chicago, Illinois

## Abstract

**Importance:**

Time-based billing options for physicians have expanded, enabling many physicians to bill according to time spent instead of medical decision-making (MDM) level for fee-for-service outpatient visits. However, no study to date has estimated the revenue changes associated with time-based billing.

**Objective:**

To compare evaluation and management (E/M) reimbursement for physicians using time-based billing vs MDM-based billing for outpatient visits of varying lengths.

**Design, Setting, and Participants:**

This economic evaluation used 2019 billing data for outpatient E/M codes and 2021 reimbursement rates from the Centers for Medicare & Medicaid Services. Modeling of generic clinic templates was performed to estimate expected yearly E/M revenues for a single full-time physician working in an outpatient clinic using fee-for-service billing.

**Main Outcomes and Measures:**

Yearly E/M revenues for different patient visit templates were modeled. The standardized length of return patient visits was 10 to 45 minutes, and new patient visits were twice as long in duration.

**Results:**

Under MDM-based billing, increased visit length was associated with decreased E/M revenue ($564 188 for 30-minute new patient visit/15-minute return patient visit vs $423 137 for 40-minute new patient visit/20-minute return patient visit). Under time-based billing, yearly E/M revenue remained similar across increasing visit lengths ($400 432 for 30-minute new patient visit/15-minute return patient visit vs $458 718 for 40-minute new patient visit/20-minute return patient visit). Compared with time-based billing, MDM-based billing was associated with higher E/M revenue for 10- to 15-minute return patient visits ($400 432 vs $564 188). Time-based billing was associated with higher E/M revenue for return patient visits lasting 20 minutes or longer. The highest modeled E/M revenue of $846 273 occurred for 10-minute return patient visits under MDM-based billing.

**Conclusions and Relevance:**

Results of this study showed that the relative economic benefits of MDM-based billing and time-based billing differed and were associated with the length of patient visits. Physicians with longer patient visits were more likely to experience revenue increases from using time-based billing than physicians with shorter patient visits.

## Key Points

**Question:**

How does the 2021 change in evaluation and management services guidelines, which allow for time-based billing inclusive of work before and after outpatient visits, affect reimbursement of physicians?

**Findings:**

In this economic evaluation of patient visits of different lengths, the medical decision-making billing method was associated with higher reimbursement for return patient visits lasting 10 or 15 minutes. For longer visits, the time-based billing method was associated with higher reimbursement.

**Meaning:**

Findings of this study suggest that the time-based billing is associated with economic benefits for physicians in lower-volume clinics with longer patient visits.

## Teaser

This economic evaluation estimates annual revenue for different durations of patient encounters among physicians in outpatient clinics.

## Introduction

In the US, physicians still receive most of their reimbursement for outpatient visits through the fee-for-service model.^1^ Within the fee-for-service model, evaluation and management (E/M) services guidelines have been used for decades to establish the level at which physicians can bill patient encounters.^2^ Under this system, a *Current Procedural Terminology* (*CPT*) code can be selected to ascertain reimbursement for a given encounter according to medical decision-making (MDM) levels.^3^ Levels of MDM, in turn, are based on the number and complexity of problems addressed at the encounter.^4^ However, studies show that physicians spend substantial time doing work that is not explicitly reportable by the E/M system of MDM-based billing, including medical record review, documentation, and coordination of care.^5,6^ As a result, many physicians report averaging 1 to 2 hours of unreimbursed, after-hours work daily.^6,7,8^ After-hours work is especially common for primary care physicians (PCPs) and has frequently been associated with increased rates of burnout.^9,10,11^

In addition to MDM-based billing, physicians can bill on the basis of visit length. Historically, time-based billing has counted only time spent face-to-face with patients.^4^ However, substantial changes to time-based billing occurred in the 2021 E/M guidelines. The 2021 guidelines allow physicians to bill for face-to-face time and for previously unreimbursed time spent on medical record review, documentation, and coordination of care on the day of the patient encounter.^3,12^ Because time-based billing monetizes previously unreimbursed services, it offers physicians an opportunity to increase revenue, compared with MDM-based billing, which still does not reimburse for these services. However, variations in patient panels and clinic schedules may be factors in different lengths of an average patient visit.^13,14^ In turn, individual physicians are likely to see different outcomes associated with these changes in billing. Changes to the economic incentives for different visit lengths could have downstream implications for clinic scheduling and patient access.

In this study, we aimed to compare E/M reimbursement for physicians using time-based billing vs MDM-based billing for outpatient visits of varying lengths. Specifically, to identify the economic incentives of expanded time-based billing for E/M revenue in different practices, we performed modeling of the expected E/M revenue for a single physician working in a primary care clinic. We then performed sensitivity analyses to illustrate how these billing changes altered the incentives for specialty physicians as well. We hypothesized that physicians with longer encounters would benefit the most from time-based billing.

## Methods

The University of Chicago Institutional Review Board deemed this economic evaluation to be nonhuman participant research and thus exempt from approval and the requirement for informed consent. We followed the Consolidated Health Economic Evaluation Reporting Standards (CHEERS) reporting guideline.

The modeling of yearly E/M revenues for an individual full-time physician compared MDM-based billing revenue with time-based billing revenue. We defined full-time work as 8 hours a day of seeing patients for 220 days a year. We limited the analysis to new and return outpatient visits with *CPT* codes 99202 to 99215, which represent the codes physicians can use for time-based billing.^4^ To calculate the proportion of new and return visits seen by PCPs, we used 2018 National Ambulatory Medical Care Survey (NAMCS) summary data.^15^ We then assumed that the physician in the model matched the proportions from the NAMCS data, with 8.5% new patient visits and 91.5% return patient visits.^15^ We also assumed that the physician scheduled twice as much time to see new patients as return patients. These assumptions allowed us to construct yearly schedules for physicians to see patients at different time intervals.

The shortest patient visit template gave physicians 20-minute visits with new patients and 10-minute visits with return patients. We analyzed schedules at regular-length visits until the longest duration, which gave physicians 90 minutes for new patient visits and 45 minutes for return patient visits. From these schedules, we calculated the number of new and return patient visits that a physician seeing patients at each time interval would have per year. Although physicians can specify MDM-based billing or time-based billing for individual patients, the physician in the model used the same billing modality for all visits to enable a comparison of the maximal incentives offered by each billing method.

### MDM-Based Billing

To calculate MDM-based billing revenue, we used Centers for Medicare & Medicaid Services (CMS) data to estimate the proportion of outpatient visits with *CPT* codes 99201 to 99215 before the addition of time-based billing.^16^ Although time-based billing was not added until 2021, we used 2019 CMS billing data to avoid any possible short-term implications of the COVID-19 pandemic.^17^ The use of 2019 CMS billing data also ensured that any billing changes associated with the expansion of time-based billing did not alter the MDM-billing distribution.^18^

**Figure 1.**
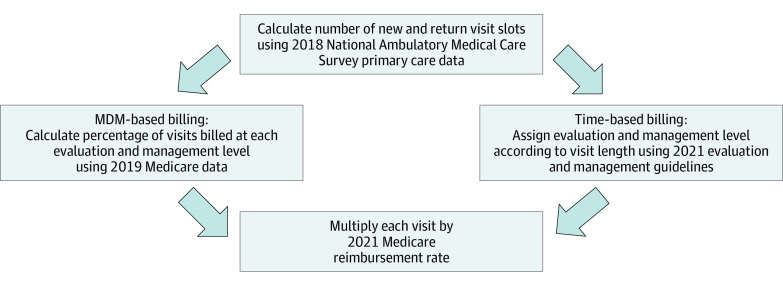
Model Schematic MDM indicates medical decision-making.

We used *CPT* codes 99201 to 99215 billed to CMS in 2019 by family medicine and internal medicine practitioners (representing approximately 73 million visits) to calculate the percentage of encounters billed at each E/M level under MDM-based billing. We assumed that the PCP in the model would match this billing distribution. By multiplying the number of new and return patient visits by the proportion of visits billed at each rate, we estimated the yearly number of visits billed at each E/M level. We then multiplied this yearly number by the 2021 CMS national nonfacility price reimbursement rate for each of the *CPT* codes (99201-99215) to arrive at the total yearly revenue (Figure 1).^19^

The *CPT* code 99201 for level 1 new patient visits was retired between 2019 (when the billing data we used were collected) and 2021 (when time-based billing was expanded).^4^ The code represented less than 0.4% of new patient visits and was used for new patient visits that could not meet level 2 billing criteria.^4,16^ Because the code no longer exists, we assigned it a value of $0, limiting the analysis to *CPT* codes 99202 to 99215.

### Statistical Analysis

#### Time-Based Billing

We assigned *CPT* codes to each visit according to the length of the encounter, including qualifying non–face-to-face time such as preparing for and documenting the encounter, as outlined in the 2021 E/M services guidelines.^4^ The 2018 NAMCS data were used to identify the breakdown of new and return patient visits. As with MDM-based billing, with time-based billing, the 2021 CMS nonfacility price reimbursement value was assigned to each *CPT* code. By multiplying the reimbursement for each visit by the number of total visits scheduled for the year, we calculated total yearly revenue.

#### Conversion Factor

Time-based billing, but not MDM-based billing, allows physicians to receive reimbursement for the non–face-to-face tasks that consume a substantial portion of the clinic day.^5^ Many physicians also spend varying lengths of time performing these tasks before or after clinic.^6,7,8^

To standardize these differences, the model constrained the physician’s clinic day to a total of 8 hours of both patient-facing and non–face-to-face tasks. Within this 8-hour day, we assumed a physician using time-based billing consistently performed reimbursable work. However, a physician using MDM-based billing who was performing the same work would have time that was not reimbursed. To account for this discrepancy, we conducted a literature review to estimate the percentage of a physician’s day spent on tasks reimbursed under time-based billing but not under MDM-based billing. We found evidence that, on average, physicians spend approximately 3 minutes before each patient visit and 4.5 minutes after each patient visit, for a total of 7.5 minutes per visit on tasks that are not reimbursed under MDM-based billing.^7,20,21^ Data from NAMCS showed that a PCP spends a mean (SD) 20.9 (0.4) minutes of face-to-face time with each patient, suggesting a total of 28.4 minutes per patient.^15^ From these calculations, we assumed that only 74% (20.9 minutes divided by 28.4 minutes) of a physician’s time under MDM-based billing was reimbursable. Thus, we multiplied all revenues from MDM-based billing by a conversion factor of 0.74.

#### Sensitivity Analysis

The base-case analysis (Table 1) assumed that the physician in the model matched the billing rates from family medicine and internal medicine practitioners in the CMS data set. To extend the analysis to other specialties, we ran sensitivity analyses examining the implications of specialty-specific E/M billing distributions for the model. We chose dermatology as a representative specialty that, on average, billed at a much lower E/M level than primary care. Cardiology was selected as a representative medical specialty that tended to bill at higher E/M levels than primary care.^16^

**Table 1.** Model Inputs and Sample Calculations for Total Revenue

	Value
Base-case model inputs	
Length of clinic day, h	8
No. of days worked per year	220
% New patient visits	8.5
% Return patient visits	91.5
Sample calculation of MDM-based billing revenue^a^	
Length of new visit, min	30
Length of return visit, min	15
No. of new visits per year	553
No. of return visits per year	5934
% Return visits billed at *CPT* code 99211	1.64
No. of return visits billed at *CPT* code 99211	97
Billing rate of *CPT* code 99211 visits, $	23.03
Yearly revenue from *CPT* code 99211 visits, $	2236
Sample calculation of time-based billing revenue	
Billing code assigned to 15-min return visits	99212
Billing rate of *CPT* code 99212 visits, $	56.88
Yearly revenue from return visits, $	337 537

Abbreviations: *CPT*, *Current Procedural Terminology*; MDM, medical decision-making.

^a^ For MDM-based billing, the calculation to obtain total revenue for visits billed at *CPT* code 99211 is shown; this revenue was added to the revenue calculated from all other visit levels for new and return patients to arrive at the total yearly revenue value.

In addition, we used NAMCS data to calculate the relative proportions of new and return patients seen by specialists, who had a higher fraction of new patient visits than PCPs (23% vs 9%).^15^ We performed a sensitivity analysis adjusting the value of the conversion factor used to account for work not reimbursed under MDM-based billing given that past studies have found physicians spend different lengths of time on unreimbursed tasks.^20,21,22^ We also reran the base-case scenario using facility price reimbursement values. All statistical calculations and plots were performed with Excel (Microsoft Corp).

## Results

**Figure 2.**
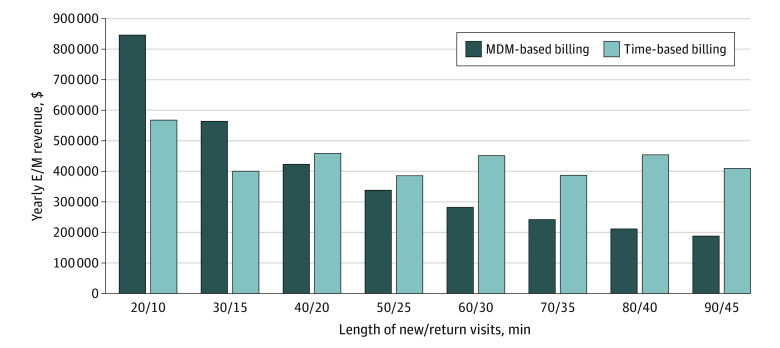
Evaluation and Management (E/M) Revenue by Visit Length New visits are assumed to always be twice as long as return visits. MDM indicates medical decision-making.

The yearly E/M revenue in the model varied inversely with the length of patient visits for MDM-based billing (Figure 2). The shortest patient visit (20-minute new patient visits, and 10-minute return patient visits) was associated with the highest E/M revenue ($846 273) (Table 2). Yearly E/M revenue decreased with each successive increase in patient visit length ($564 188 for 30-minute new patient visit/15-minute return patient visit vs $423 137 for 40-minute new patient visit/20-minute return patient visit), with the longest visits (90-minute new patient visits, and 45-minute return patient visits) showing the lowest E/M revenue ($188 065).

**Table 2.** Sensitivity Analyses for Yearly Revenue

Length of return/new visit, min	Revenue, $								
	Base-case scenario		Specialty proportion of new visits^a^		Conversion factor adjustments for MDM-billing revenue^b^			Specialty billing distribution adjustments for MDM-billing revenue^c^	
	Time-based billing revenue	MDM-based billing revenue	Time-based billing revenue	MDM-based billing revenue	Conversion factor = 1	Conversion factor = 0.85	Conversion factor = 0.65	Cardiology	Dermatology
10/20	567 649	846 273	523 226	775 512	1 149 960	977 466	747 474	972 048	575 697
15/30	400 432	564 188	400 423	517 009	766 648	651 651	498 321	648 039	383 802
20/40	458 718	423 137	418 978	387 757	574 980	488 733	373 737	486 024	287 849
25/50	385 614	338 511	378 910	310 205	459 986	390 988	298 991	388 821	230 280
30/60	451 310	282 094	437 150	258 504	383 324	325 825	249 161	324 019	191 901
35/70	386 832	241 792	374 700	221 575	328 559	279 275	213 563	277 727	164 484
40/80	454 172	211 568	414 533	193 878	287 489	244 366	186 868	243 011	143 924
45/90	409 894	188 066	382 960	172 336	255 554	217 221	166 110	216 017	127 936

Abbreviation: MDM, medical decision-making.

^a^ Represents the outcome of changing the frequency of new visits to match the frequency in specialty clinics.

^b^ Represents the revenue for MDM-based billing using different conversion factors to account for unreimbursed work. A conversion factor of 1 represents the physician using 100% of their time doing reimbursable tasks.

^c^ Represents the adjusted frequency of each *Current Procedural Terminology* 99202 to 99215 billing code vs the frequencies used in cardiology and dermatology but with the same ratio of new to return patients as in the base-case scenario.

Unlike with MDM-based billing, the E/M revenue in the model remained relatively similar across visit lengths ($400 432 for 30-minute new patient visit/15-minute return patient visit vs $458 718 for 40-minute new patient visit/20-minute return patient visit). Similar to MDM-based billing, the highest E/M revenue ($567 649) was associated with 20-minute new patient visits and 10-minute return patient visits. The lowest E/M revenue ($385 614) was associated with 50-minute new patient visits and 25-minute return patient visits (Table 2).

In the model, the revenue advantage of time-based billing over MDM-based billing increased with longer visits. For shorter visits (20-30 minutes for new patient visits, and 10-15 minutes for return patient visits), MDM-based billing was associated with higher revenues compared with time-based billing (20-minute new patient visits and 10-minute return patient visits: $846 273 vs $567 649). Starting at 40-minute new patient visits and 20-minute return patient visits, time-based billing, compared with MDM-based billing, was associated with higher E/M revenues ($458 718 vs $423 137).

We found that MDM-based billing revenue was sensitive to the E/M billing distribution used. Substituting cardiology’s billing distribution of higher mean E/M levels compared with primary care was associated with a 15% increase in all E/M revenues for MDM-based billing across visit lengths (eg, from $423 137 to $486 024 for 40-minute new patient visits and 20-minute return patient visits) (Table 2). This shift played a role in time-based billing compared with MDM-based billing maximizing E/M revenue only when new patient visits were 60 minutes or longer and when return patient visits were 30 minutes or longer. In contrast, using dermatology’s lower E/M billing distribution was associated with a 32% decrease in all E/M revenues for MDM-based billing across visit lengths (from $423 137 to $287 849 for 40-minute new patient visits and 20-minute return patient visits). This shift played a role in time-based billing compared with MDM-based billing having greater E/M revenue starting at 30-minute new patient visits and 15-minute return patient visits. Table 2 shows that MDM-based revenue results were sensitive to the conversion factor used to account for unreimbursed work in MDM-based billing. We found that MDM-based revenue increased by 36% across visit lengths when the conversion factor was increased to 1 (from $423 137 to $574 980 for 40-minute new patient visits and 20-minute return patient visits), and MDM-based revenue decreased by 12% when the conversion factor was decreased to 0.65 (from $423 137 to $373 737 for 40-minute new patient visits and 20-minute return patient visits). Increasing the percentage of new patient visits to the 23% new patient rate of specialty physicians affected all E/M revenue calculations by less than 10% (Table 2). For this higher proportion of new patient visits, time-based billing was associated with more revenue than MDM-based billing starting at 40-minute new patient visits and 20-minute return patient visits ($418 978 vs $387 757). Using facility price reimbursement levels was associated with lowered E/M revenues globally without affecting the previously noted association between MDM-based billing and time-based billing (eTable in the Supplement).

## Discussion

A variety of factors were associated with the length of patient visits, but any clinic must consider economic incentives to maintain its financial viability. The underlying hypothesis that physicians change their billing practices in response to shifting billing incentives is already supported by data, such as a recent study reporting that physicians began billing at higher levels just after the expansion of time-based billing.^18^ In the present economic evaluation, the models suggested that E/M revenue from MDM-based billing was associated with the number of patients seen per hour, incentivizing shorter patient visits. Conversely, we found that time-based billing removed the association between patients seen per hour and revenue, allowing physicians to have longer patient visits without a loss of E/M revenue. In this modeling, shorter visit lengths were associated with MDM-based billing that earned more revenue, although we acknowledge that physicians are unlikely to bill higher levels of MDM with extremely short visits. As clinic visits became longer, time-based billing became the revenue-maximizing strategy. Moreover, MDM-based billing and time-based billing yielded the most similar revenues in the model for 40-minute new patient visits and 20-minute return patient visits. This visit length in the model was associated with reported mean visit lengths in actual practice, suggesting that time-based billing has limited implications for many clinics.^15,18,23^

The highest E/M revenues in this study were associated with a combination of short patient visits and MDM-based billing. This finding demonstrates that time-based billing is unlikely to change financial incentives given for shorter visits.^24,25^ However, physicians with lower volume and longer patient visits can benefit from time-based billing in multiple ways. Because the models showed E/M revenue was greater with time-based billing at longer visits, physicians with longer patient visits were more likely to gain a revenue increase from the time-based billing option than physicians who scheduled shorter patient visits. In addition, because there was no association between E/M revenue and visit length under time-based billing, physicians with longer patient visits could further extend their patient visit length without a noticeable decrease in E/M revenue. Previous studies have shown that physicians with time constraints are less likely to complete preventive medicine tasks.^26,27^ Therefore, the flexibility in patient scheduling afforded by time-based billing could help physicians better address preventive medicine.^28^ A decrease in patients per hour could also be used to help physicians complete non–face-to-face tasks, such as documentation, that traditionally have been pushed to after hours, potentially contributing to decreased physician burnout.^29,30^ At the national level, longer patient visits with a fixed health care workforce could be a factor in limited patient access to their physicians. Moreover, by reimbursing only physician time, time-based billing may penalize efficient physicians and team-based clinic workflows and reward inefficiencies while increasing health care costs.

High-volume and low-volume clinics are often located in different areas and serve different patient populations.^8,9^ As such, the finding that time-based billing is less advantageous for high-volume clinics than low-volume clinics could have implications for health equity. As a corollary, high-volume, low-acuity specialties may be less likely to benefit from time-based billing.^31^

Downstream sources of revenue and the health care system within which a clinic operates were factors in a clinic’s scheduling, suggesting that E/M revenue does not exist in a vacuum. Similarly, individual physicians affiliated with a large health care system may react more directly to economic incentives affecting their personal earnings, not the clinic’s overall revenue.^32^ Still, previous studies have found that clinics respond to economic incentives.^14,18,32,33^ More research is needed to better understand the complex economic associations between outpatient scheduling and billing incentives.

### Strengths and Limitations

This study has some strengths. The findings are generalizable to different specialties and clinics. The study reported yearly E/M revenue for a full-time physician, but the relative difference between MDM-based billing revenue and time-based billing revenue was unchanged for physicians not working a 40-hour work week. By incorporating data on after-hours documentation, we also accounted for the much longer work hours actually spent by many physicians who are scheduled to be in clinic for 40 hours a week.^6,7,8^ Although the base-case scenario used PCP billing data, the analysis can be readily repeated for specialty or even clinic-specific data. For example, we used previously published work to estimate the mean time spent on unreimbursed tasks per patient, but physicians can substitute individual data to obtain a personalized estimate.

This study also has some key limitations. First, we used Medicare data to identify the distribution of *CPT* codes for MDM-based billing. If Medicare beneficiaries required more MDM than patients without Medicare coverage, then use of Medicare data artificially increased the MDM-based billing revenues. We also were unable to account for the implications of recent changes to simplify MDM-billing guidelines because the MDM billing distribution in the model used 2019 data.

Second, we assumed that physicians used either MDM-based billing or time-based billing for all of their patient encounters. In actual practice, a physician can choose whichever billing method can generate a higher reimbursement.^4^ Similarly, in the model, the calculations held constant the E/M billing distribution for MDM across different lengths of visits. In practice, short patient visits are more likely to be coded at lower E/M levels, potentially contributing to MDM-based revenue being artificially high at shorter visits. Furthermore, longer visits are more likely to be coded at higher E/M levels, which could be associated with MDM-based revenue calculations being lower for longer visits.

Third, the E/M revenue model excluded services other than patient visits with *CPT* codes 99201 to 99215 and thus did not consider other sources of revenue, such as preventive health visits or procedures. The model also did not consider downstream revenue associated with ancillary services (eg, laboratory testing and diagnostic imaging) or referrals made during visits. The financial value of these services and referrals can be much greater than the E/M revenue associated with direct patient visits.^34^ Downstream revenue is likely to vary greatly between specialties and even practices within a specialty but regardless serves as an economic argument against longer patient visits. Even if time-based billing allows a physician to not lose direct E/M revenue with longer patient visits, fewer visits may ultimately be a factor in decreased downstream revenue. For example, PCPs affiliated with a large health care system generate referrals to that system’s specialists, providing a source of revenue that goes well beyond the individual physician. Under advanced alternative payment models, such as global capitation, revenue is disconnected from billing regardless of visit length.^35,36,37^ Physicians using these reimbursement systems are unaffected by time-based billing.^38^

## Conclusions

In this economic evaluation, we reported yearly E/M revenue earned exclusively through MDM-based billing or time-based billing for an individual physician receiving 2021 CMS nonfacility price reimbursement rates. The economic benefits of MDM-based billing and time-based billing were associated with the length of patient visits. Using time-based billing, physicians with longer patient visits were more likely to experience revenue increases than physicians with shorter patient encounters. Possible future changes to billing regulations may have similar implications for physicians’ economic incentives. Further studies using clinic- or system-level data may clarify the association of indirect and downstream revenue with the economic incentives offered by time-based billing.

## Notes

Supplement. **eTable.** E/M Revenue Calculated with Facility Price Payments
