# Supplementary material for: Association of Time-Based Billing With Evaluation and Management Revenue for Outpatient Visits
Source: JAMA Netw Open. 2022 Aug 31;5(8):e2229504. doi: 10.1001/jamanetworkopen.2022.29504 (PMC9434360; doi:10.1001/jamanetworkopen.2022.29504)
Supplement: Supplement. — eTable. E/M Revenue Calculated with Facility Price Payments [file jamanetwopen-e2229504-s001.pdf]

Supplemental Online Content

Miksanek TJ, Edwards ST, Weyer G, Laiteerapong N. Association of time-based billing with evaluation and management revenue for outpatient visits. *JAMA Netw Open*. 2022;5(8):e2229504. doi:10.1001/jamanetworkopen.2022.29504

eTable. E/M Revenue Calculated with Facility Price Payments

This supplemental material has been provided by the authors to give readers additional information about their work.

© 2022 Miksanek TJ et al. *JAMA Network Open.*

eTable. E/M Revenue Calculated with Facility Price Payments

Length of Visit (Minutes)	Time-Based Billing Revenue	MDM-Based Billing Revenue
10 Return, 20 New	$364,411	$643,396
15 Return, 30 New	$262,041	$428,935
20 Return, 40 New	$337,835	$321,698
25 Return, 50 New	$287,865	$257,360
30 Return, 60 New	$349,773	$214,468
35 Return, 70 New	$299,802	$183,827
40 Return, 80 New	$409,894	$160,849
45 Return, 90 New	$352,406	$142,981

© 2022 Miksanek TJ et al. *JAMA Network Open.*
